# Reviewing the causes of electrocardiographic pauses

**DOI:** 10.5830/CVJA-2017-041

**Published:** 2017

**Authors:** Charle Viljoen, Ashley Chin, Robert Smith

**Affiliations:** Division of Cardiology, Groote Schuur Hospital and the University of Cape Town, South Africa; Division of Cardiology, Groote Schuur Hospital and the University of Cape Town, South Africa; Department of Medicine, Groote Schuur Hospital and the University of Cape Town, South Africa

**Keywords:** ECG, sinus node dysfunction, SA exit block, sinus arrest

## Abstract

The electrocardiographic term ‘pause’ refers to the prolonged R-R interval that represents the interruption in ventricular depolarisation. This article presents a case of sinus node dysfunction and provides a diagnostic approach to pauses on the ECG.

## Introduction

A 48-year-old man was referred to the Cardiac Clinic at Groote Schuur Hospital for evaluation of suspected symptomatic aortic stenosis. He had a medical history of hypertension, which was well controlled on amlodipine 10 mg and atenolol 50 mg once daily.

He had presented with three episodes of syncope in the three months prior to assessment. The syncope was not related to exertion, standing or other specific situations and occurred without any prodrome. He was not troubled by any dyspnoea, and he denied chest pain and any palpitations.

Examination excluded severe aortic stenosis. He had regular, good volume pulses, a normal jugular venous pressure and an undisplaced apex beat with normal character. There was a soft ejection systolic murmur, best heard at the lower left sternal border with no radiation. The lung bases were clear. An electrocardiogram (ECG) and echocardiography were performed. Echocardiography showed a normal aortic valve with no evidence of aortic stenosis.

**Fig. 1.**
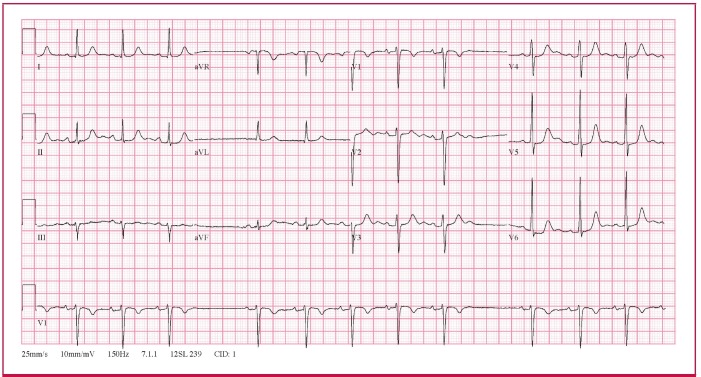
The 12-lead ECG is in keeping with sino-atrial exit block.

The ECG (Fig. 1) showed an irregular rhythm with intermittent pauses. There were no premature complexes preceding the pauses. However, during each of the pauses, there were no P waves at the expected time interval. The R-R interval during the pause was twice the R-R interval before and after the pause. Similarly, the P-P interval during the pause was twice that of the P-P interval before and after the pause. These ECG features are in keeping with sino-atrial (SA) exit block.

**Fig. 2.**
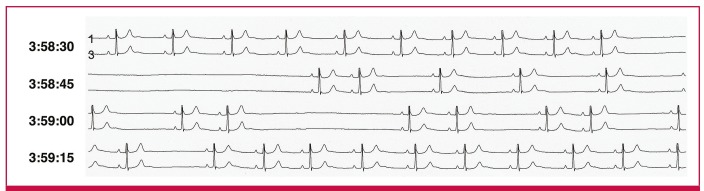
A 24-hour Holter ECG showing an episode of sinus arrest lasting seven seconds (starting just before 3:58:45) and intermittent sino-atrial exit block (just after 3:59:15).

A 24-hour Holter ECG was subsequently done (Fig. 2), which showed intermittent SA exit block and an episode of sinus arrest lasting seven seconds, which did not trigger any escape beats. The patient was diagnosed with symptomatic sinus node dysfunction and an AAIR pacemaker was implanted. The patient has been asymptomatic since.

## Causes of pauses

**Fig. 3.**
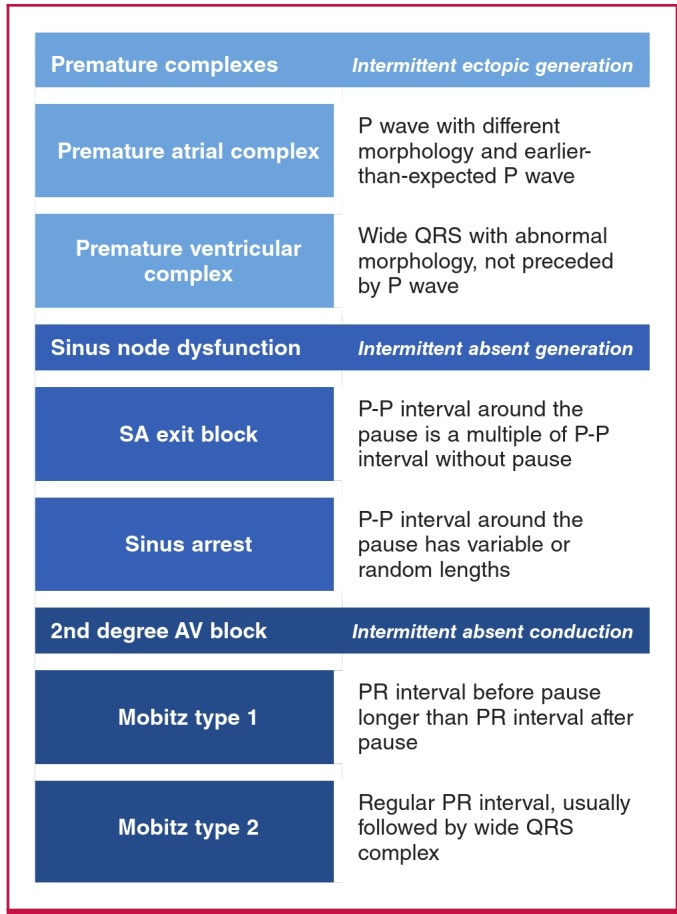
A diagnostic approach to electrocardiographic pauses.

The electrocardiographic term ‘pause’ refers to the prolonged R-R interval that represents the interruption in ventricular depolarisation. The differential diagnosis of a pause with the characteristic feature of each is shown in Fig. 3.

**Fig. 4.**
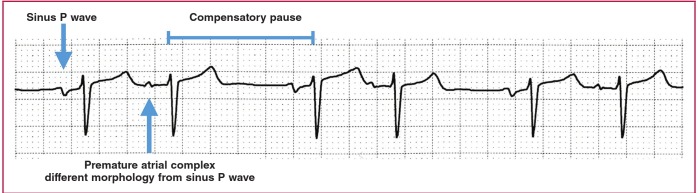
Premature atrial complex with compensatory pause.

**Fig. 5.**
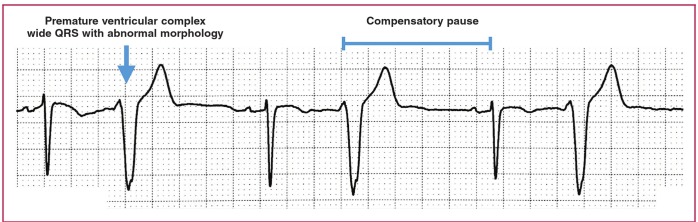
Premature ventricular complex with compensatory pause.

In the presence of a pause, one should exclude premature complexes with compensatory pause. If the ectopic beat failed to reset the sinus node, the premature complex would be followed by a compensatory pause, i.e. the R-R interval after the premature complex is longer than the R-R interval between normal sinus beats. A premature atrial complex is recognised as an early P wave with a different morphology from the sinus P wave (Fig. 4), and a premature ventricular complex is the wide QRS complex with abnormal morphology that is not preceded by a P wave (Fig. 5).

In the absence of premature complexes, one should determine whether the pause is caused by intermittent absent impulse generation (i.e. sinus node dysfunction) or intermittent absent impulse conduction [i.e. second-degree atrio-ventricular (AV) block].^1^ Sinus node dysfunction refers to the pause in atrial depolarisation, which is either caused by sinus arrest or SA exit block.^2^ The hallmark of sinus node dysfunction is missing P waves on the 12-lead ECG.^3^

**Fig. 6.**
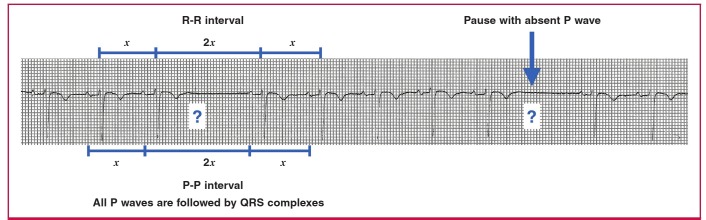
Sino-atrial exit block.

In SA exit block, the atria fail to depolarise after the SA node discharges, because the impulse cannot leave the SA node. Because the atria do not depolarise, there is no P wave visible on the ECG tracing each time the impulse fails to leave the SA node. The SA node discharge is too small to be seen on a 12-lead ECG, therefore there is no waveform visible during the SA exit block. The P-P interval during SA exit block is a multiple of the normal P-P interval, because when P waves appear, they occur at their scheduled time (Fig. 6).^1^,^3^

**Fig. 7.**
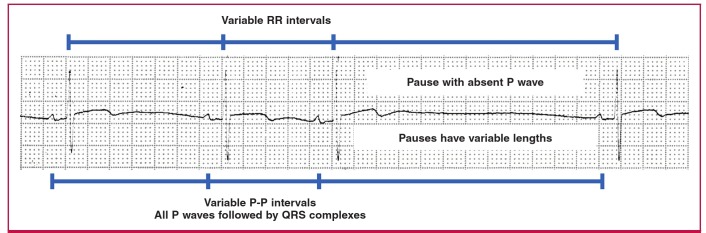
Sinus arrest with no escape beats.

In sinus arrest, the SA node does not discharge. If there is no other atrial ectopic that takes over as a pacemaker, there will be no atrial depolarisation, and therefore no P waves will be visible on the ECG for the duration of the sinus arrest. If sinus arrest is long enough, an escape beat or escape rhythm may be triggered, which will manifest as QRS complexes that are not preceded by P waves. In sinus arrest, the pause duration can be variable, and the P-P interval is therefore not necessarily a multiple of the normal R-R interval (Fig. 7).^1^

In second-degree AV block, the atrial depolarisation intermittently fails to conduct to the ventricles. On the 12-lead ECG, this will manifest as intermittent absent QRS complexes, i.e. more P waves than QRS complexes.

**Fig. 8.**
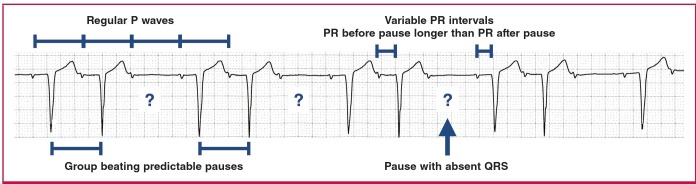
Mobitz type 1 second-degree AV block (Wenckebach).

Mobitz type 1 second-degree AV block manifests with group beating, with variable PR intervals. The PR interval typically increases in length until the pause, with the PR interval after the pause shorter than the PR interval before the pause (Fig. 8).

**Fig. 9.**
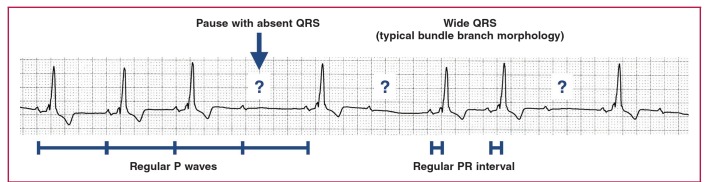
Mobitz type 2 second-degree AV block.

Mobitz type 2 second-degree AV block has constant PR intervals, with unpredictable loss of conduction of P waves not followed by QRS complexes. The QRS complexes in Mobitz type 2 are typically wide with typical bundle branch morphology (Fig. 9).

## Management of sinus node dysfunction

Management of sinus node dysfunction depends on whether the patient is experiencing symptoms or not. Whereas asymptomatic patients do not require treatment, patients who are symptomatic are treated by insertion of a permanent pacemaker. Pacemaker therapy does not prolong life but relieves symptoms.^4^
